# Youth paying for sex: what are the associated factors? Findings from a cross-sectional study in Cambodia

**DOI:** 10.1186/s12889-017-4999-8

**Published:** 2018-01-08

**Authors:** Soaman Dizechi, Carinne Brody, Sovannary Tuot, Chhorvann Chhea, Vonthanak Saphonn, Kunthearith Yung, Sanh Kim, Siyan Yi

**Affiliations:** 1 Center for Global Health Research, College of Education and Health Sciences, Touro University California, Vallejo, USA; 2 KHANA Center for Population Health Research, No 33, Street 71, Tonle Bassac, Chamka Mon, Phnom Penh, Cambodia; 3 National Institute of Public Health, Phnom Penh, Cambodia; 4 University of Health Sciences, Phnom Penh, Cambodia; 5 Department of School Health, Ministry of Education, Youth and Sports, Phnom Penh, Cambodia

**Keywords:** Paying for sex, HIV, Youth, Risk factors, Cross-sectional survey, Cambodia

## Abstract

**Background:**

At-risk male youth in Cambodia who purchase sex are at greater risk for HIV compared to the general population. Factors associated with paying for sex among youth are poorly studied, both globally and in Cambodia. This study aimed to identify specific factors associated with transactional sex with women among most-at-risk male youth in Cambodia.

**Methods:**

This cross-sectional questionnaire survey was conducted with 405 sexually active male youth aged 16–24 recruited at ‘hotspots’ in the capital city of Phnom Penh and seven provinces. We collected data on demographic factors, sexual behaviors, HIV testing and other potential factors. Multivariable logistic regression analysis was used to identify factors associated with transactional sex.

**Results:**

In total, this study included 405 male youth with a mean age of 21.3 (SD = 2.2). Of the total respondents, 82.5% (*n* = 334) have ever paid for sex. After controlling for potential confounding, participants who purchased sex in the last 12 months remained significantly more likely to be older than 18 (AOR = 3.60, 95% CI = 1.26–10.62), reside in an urban area (AOR = 2.29, 95% CI = 1.24–4.20), never have been married (AOR = 9.58, 95% CI = 4.34–21.12), spend less than 2.55 USD per day (AOR = 2.22, 95% CI = 1.12–4.40), and have had more than 4.6 sexual partners in the past year (AOR = 16.73, 95% CI = 4.71–59.36).

**Conclusions:**

This study highlights the high proportion of Cambodian male youth who paid for sex and the potential challenges to addressing this issue. While the majority of HIV prevention interventions surrounding sex work are aimed at female sex workers themselves, targeting the demand side of sex work, particularly the local demand, may be an important next step towards a sustainable HIV prevention.

## Background

Globally, the proportion of men who pay for sex varies widely among cultures and countries [1]. In one analysis based on 21 studies from across the globe, Cambodia appears to be the country with the highest proportion of men from the local population who pay for sex [1]. Estimates in Europe range from 7% in the United Kingdom to 45% in Italy, while estimates in Asia range from 6.4% in China to 80% in Cambodia. A 2004 Behavioral Surveillance Survey in Cambodia found that between 1997 and 2003, 59–80% of moto-taxi drivers, military and police men, occupational groups who are known to be common clients of sex workers, report ever having had sex with a sex worker [2].

Across the world, factors that are associated with paying for sex among men also range broadly and are not well studied [3]. One estimate from 1991 suggested that only 1% of all research on prostitution addressed the demand side of sex work, underscoring a substantial knowledge gap in factors associated with the demand side of the sex-industry [4].

From the limited studies that are available on demand-side factors, there are several ethnographic studies and small survey-based research in Western countries which suggest that older age, lower educational attainment [5], being single, being on disability pension, having had an early sexual debut, reporting a high number of sexual partners [6] and reporting risky sexual practices such unprotected sex [7] were associated with paying for sex among men. However, other studies from Western countries argue that men who purchase sex are a heterogeneous group of all ages, socioeconomic status levels, occupation and ethnic/racial groups [8, 9].

Additionally, studies in Western countries suggested that there exists personal reasons that increase the likelihood that men report ever paying for sex such as a satisfying sexual needs, desire for particular sexual acts or practices that regular partners are unable or unwilling to accommodate, sexual relief, convenience, entertainment and the belief that people are not meant to be monogamous. Less common factors have included influence of alcohol and drugs, companionship, avoiding relationship and feelings of personal inadequacy according to accepted social norms relating to masculinity [5, 10–13].

In Asian countries, the forces at play may be drastically different. Qualitative work has highlighted the importance of cultural influences for why men pay for sex in Asian countries. An International Organization for Migration (IOM) study found that it was common for men from Japan, India and Thailand to report that their first experience with a sex worker was arranged by friends, that they plan to take their sons to sex worker when they reached the age of 17 and that paying for sex was part of the social demands of masculinity in their cultures [14].

Cambodia has been recognized for successful HIV prevention and control efforts that have reduced HIV prevalence in the general adult population from 1.7% in 1998 to 0.7% in 2013 [15, 16]. During its peak in 1998, the National Center for HIV/AIDS, Dermatology and STD (NCHADS) reported 52.1% of direct female sex workers, women whose main source of income is generated through sex work, and 19.5% of indirect female sex workers, women who work in entertainment venues and make supplementary income through sex work but do not necessarily identify as sex workers, tested positive for HIV [17]. More recent Cambodian HIV Sentinel Surveys show that from 1998 to 2006, HIV prevalence rates among direct and indirect female sex workers have fallen to 14.7% and 9.3%, respectively [17]. In 2016, HIV is concentrated among certain populations at higher risk of HIV infection such as female entertainment workers (FEWs), people who inject drugs (PWID), transgender women (TG) and men who have sex with men (MSM) [18]. Many of the individuals that make up these high-risk groups are under 25 years old [16]. Most recent estimates show Cambodia has the estimated youngest population in Southeast Asia with 19.1% of the population comprising young people between 15 and 24 years of age [19]. National and international governing bodies have made concerted efforts to understand the pathways of risk for young people in order to develop and implement tailored prevention strategies [18]. An area that has received little attention is the risk factors and behaviors associated with male youth (16–24) who pay for sex.

In 2008, in an effort to comply with the United Nations protocol on human sex trafficking and to reduce HIV by eliminating brothels, the government of Cambodia passed the Cambodian legislation ‘Law on Suppression of Human Trafficking and Sexual Exploitation,’ effectively banning third party procurement of sex (i.e. brothels and pimps). Prostitution itself is still legal in Cambodia [20]. As a result, direct sex workers, both male and female, have transitioned into more underground and hidden street-based or entertainment establishments to sell sex [18]. In addition, more young women are engaging in indirect methods of selling sex through entertainment venues such as a bar, nightclub, karaoke parlor, massage parlor, sauna and private party or strip clubs [20–22]. Changes in the sex work environment may be making it easier for male youth in Cambodia to access and/or pay for sex with female sex workers because they are accessed through more accessible venues.

In Cambodia, few studies have specifically addressed the demand side of sex work. From recent national reports, known socio-demographic factors associated with men paying for sex include younger age; employment as military personnel, police officer or moto-taxi driver; living in an urban location and reporting a high number of sexual partners [23–25]. The culture of paying for sex in Cambodia has also been described as a significant part of male identity and that young men are inculcated into this cultural norm [26]. A 2004 Cambodian report demonstrated that of military, police and moto-taxi drivers ages 20–24 years old, 68.2% reported sex with a direct female sex worker in the past year, and 49.9% reported sex with a direct female sex worker in the past month [2]. Paying for sex has been described as an ‘ordinary part of Cambodian culture,’ and there is little stigma associated with men admitting that they have paid for sex [21, 27].

A recent mixed methods study of 133 Cambodian men focused on the socio-demographic composition of men who bought sex from a FEW [20]. The report states that 92% of the men reported they were with either friends or relatives when they first bought sex, and 91% of the men bought sex at least once a month or more often. Qualitatively, common reasons men stated they bought sex was ‘in order to satisfy an immediate sexual urge.’ Less frequently, men stated they bought sex because they were intoxicated, were responding to peer pressure, were bonding with male friends, wanted to learn about sex, were frustrated with current relationship and were seeking variety.

The high rates of young men paying for sex in Cambodia represents a national health concern because these young men are at higher risk for acquiring HIV and are considered a bridging population for HIV transmission to the general population [17, 23]. There exists a significant knowledge gap on the factors associated with paying for sex among youth in Cambodia despite the fact that men may be important targets for interventions that aim to decrease the health risks of paying for sex for both clients and sex workers [20]. Furthermore, while there has been a large focus on increasing condom use in the sex worker population, condom use with ‘sweethearts’ remains low, which may be a driving force behind increases in HIV and STIs in the general population [21, 28].

In addition, while many public health efforts take a harm reduction approach to addressing the negative health consequences of sex work by educating and empowering sex workers themselves, the broader context that creates a demand for sex work in the first place is rarely the focus of interventions. This study represents a step towards intervening on the demand side of the sex work industry, a strategy that has the potential to create more sustainable change. A better understanding of the different factors associated with paying for sex in at-risk young men can inform future strategies to deploy public health interventions aimed at this group. This study aims to identify specific factors associated with paying for sex with females among most-at-risk male youth in Cambodia.

## Methods

### Participants and sampling

This study draws from a larger cross-sectional survey, Cambodia’s Most at Risk Young People Survey (MARYP) conducted in 2010 [29]. The sample size for this survey was calculated based on the expected prevalence of several variables to be measured in this survey based on the National Youth Behavior Survey of 2004 [30]. Of 1312 youth approached, data were collected from 1234 participants (response rate of 94.0%) in eight city and provinces with characteristics suggesting that populations had high rates of HIV risk behaviors (Battambang, Banteay Meanchey, Kampong Cham, Siem Reap, Phnom Penh, Preah Sihanouk, Koh Kong and Svay Rieng). These characteristics included high incidences of rape, human trafficking and migration as well as high number of female sex workers and MSM. Details of this study have been published elsewhere [31, 32].

### Sampling strategy

A two-stage cluster sampling design was used. Hot spots, or times and locations where young people are likely to gather, were used as cluster units. The mapping of hot spots, carried out by a team of Cambodian youth, included the following locations: bars, karaoke parlors, massage parlors, street corners, places where youths frequently gather (football field, skating field, etc.), public parks, snooker clubs and computer game shops. A time-location method was used to recruit respondents from this fluid population at hotspots during times when most youth were on site. As such, hotspots were not geographical locations alone but were conditional on the time of the day, week and month at which the sampling took place. At each stratum, 63 clusters were selected using an equal probability sampling method. At the second stage, 20 respondents were randomly selected from each cluster. From each cluster, half of the selected respondents were aged from 10 to 19. This following analysis should not be considered a probability sample since it is a sub-sample based on sexual experience. Participants had to be male aged 10–24 years, be present in the selected hotspots, be able to communicate in Khmer, and be able to provide an informed consent to participate in the survey.

### Survey tool

Data were collected through face-to-face interviewer-administered surveys in Khmer by youth data collectors that were recruited from local NGOs using a structured questionnaire with 77 questions. All peer data collectors underwent a training, which was conducted over 4 days where trainers went over study protocol, the questionnaires, field practice and review of sampling technique, consent forms and document organization. The variables were measured using standardized tool adapted from previous studies in the same populations [30], the most recent Cambodia Demographic and Health Survey [33], as well as from other studies on risky behaviors and young people in Cambodia [34]. Socioeconomic characteristics included age, marital status, years of education completed, current school enrollment status, monthly income, monthly expenses and housing situations.

Risky sexual behaviors questions included questions about age at first sex, age and type of first sexual partner, current sexual partners, places of recruitment of sex workers if involved in paid sex, current and past relationships, condom use with sex workers and girlfriends, occurrence of sex while under the influence of alcohol or drugs and ever had an HIV test. The outcome variable was derived from a survey question that asked: ‘In the past 12 months, have you ever paid or given gifts to have sex with a woman?’

### Data analyses

Questionnaire data were entered using EpiData. All questionnaires were entered twice to minimize data entry errors. Inconsistencies were solved by reviewing the paper copy of the questionnaire. No identifying information was entered. Descriptive analyses were conducted to describe demographic characteristics and risky sexual behaviors of the study sample using mean with standard deviation (SD) for continuous variables and number (%) for categorical variables. Chi-square or Fisher’s exact test was used for categorical variables and *t*-tests were used to compare continuous demographic characteristics and risky sexual behaviors among male youth who reported having paid for sex to those among male youth who reported not having paid in the past 12 months.

A multivariate logistic regression model was constructed to examine the independent association between demographic and behavioral characteristics and risky sexual behavior and paying for sex. Our initial model was informed by the literature in terms of what we would expect to be significant regardless of significance at bivariate level. A final model was developed by removing variables with the highest *p*-value, refitting the model and repeating the step until all *p*-values of included variables were less than 0.05. Odds ratio (OR) for bivariate analyses and adjusted odds ratio (AOR) for multivariate analyses were calculated and presented with 95% confidence intervals (CI) and *p*-values. STATA version 13.1 (StataCorps LP, Texas USA) was used for all data analyses.

### Ethical consideration

The study was reviewed and approved by the Cambodian National Ethics Committee for Health Research (No. 133 NECHR). Touro University California granted exempt IRB approval. A written informed consent was sought from the study participants before the start of the survey. They were made clear that participation in this study was voluntary, and they could refuse or discontinue their participation at any time. Privacy of the respondents was protected by conducting the interviews at a private place, and no personal identifiers were collected in the questionnaires or field notes. Study participants were also informed of the option for escorted referral to appropriate existing services if they so wished. The survey was guided by a steering committee chaired by key stakeholder representatives of at-risk youth in the community. A Youth Advisory Group was also convened to inform each stage of the research design and findings.

## Results

### Socio-demographic characteristics

Table 1 depicts socio-demographic characteristics of the respondents. This study included 405 sexually active male youth within an age range of 16 to 24. Only 17 respondents were younger than 18. Of total, 82.5% (*n* = 334) reported having paid for sex in the past 12 months. Compared to respondents who reported not having paid for sex, respondents who reported having paid for sex were significantly more likely to be older than 18 (97.0% vs. 90.1%, *p* = 0.009) and to live in an urban area (75.5% vs. 56.3%, *p* < 0.001), and were significantly less likely to be married (69.0% vs. 93.7%, *p* < 0.001).

**Table 1:** Socio-demographic characteristics of Cambodian male youth who have or have not paid for sex in the last year (*n* = 405)

Socio-demographic characteristics	Having paid for sex (*n* = 334)	Not having paid for sex (*n* = 71)	
	Number (%)	Number (%)	*P*-value^*^
Have ever paid for sex (*n* = 405)	334 (82.5)	71 (17.5)	
Mean age (in years) (±SD)	21.2 (± 2.1)	21.4 (± 2.3)	0.50
Age 18 and above			0.009
Yes	324 (97.0)	64 (90.1)	
No	10 (3.0)	7 (9.9)	
Location surveyed			<0.001
Urban	252 (75.5)	40 (56.3)	
Rural	82 (25.6)	41 (43.7)	
Marital status			<0.001
Married	18 (5.4)	17 (23.9)	
Never married	313 (93.7)	49 (69.0)	
Divorced or separated	1 (0.3)	2 (4.2)	
Living with sexual partner	2 (0.6)	3 (4.2)	
Province surveyed			<0.001
Phnom Penh	93 (27.9)	7 (9.9)	
Battambang	25 (7.5)	3 (4.2)	
Kampong Cham	30 (9.0)	17 (23.9)	
Siem Reap	40 (12.0)	8 (11.3)	
Bantey Mean Chey	20 (6.0)	1 (1.4)	
Svay Rieng	27 (8.1)	6 (8.5)	
Koh Kong	62 (18.6)	13 (18.3)	
Preah Sihanouk	36 (10.8)	16 (22.5)	
Mean years of formal schooling (±SD)	9.9 (±3.9)	9.2 (±3.6)	0.21
Completed primary school	266 (79.6)	52 (73.2)	0.23
Currently enrolled in school	116 (34.7)	20 (28.2)	0.28
Occupation			0.80
Unemployed	31 (14.5)	10 (18.5)	
Seller/vendor/trader	145 (67.8)	36 (66.7)	
Manual/laborer	2 (0.9)	1 (1.9)	
Farmer	6 (2.8)	0 (0.0)	
Factory worker	4 (1.9)	1 (1.9)	
Other	26 (12.2)	6 (11.1)	
Having an income			0.18
Yes	222 (66.5)	53 (74.7)	
No	112 (33.4)	18 (25.3)	
If yes, mean monthly income (USD) (± SD)	267.6 (±454.1)	228.6 (±73.2)	0.07
Mean daily expenses in the past month (USD) (±SD)	2.43 (±2.0)	3.46 (±6.4)	0.02

*Abbreviations: SD* standard deviation, *USD* United States Dollar

^*^Chi-square test or Fisher’s exact test was used as appropriate for categorical variables and t-test was used categorical variables

The mean number of years of school completed was 9.9 among respondents who reported having paid for sex (SD = 3.9 years) and 9.2 (SD = 3.6 years) among those who reported not having paid for sex. Similarly, over 70% of both respondents who reported having paid for sex and respondents who reported not paying for sex completed at least 6 years of school (79.6% vs. 73.2%, *p* = 0.23). Monthly income for both groups of respondents varied greatly; the mean was greater for those who reported having paid for sex as compared to those who reported not having paid for sex ($267.6±454.1 vs. $228.6±473.2, *p* = 0.07). Daily expenses for the past month were significantly different between the two groups; the mean for respondents who reported having paid for sex was $2.43 (SD = 2.0), whereas respondents who reported not having paid for sex spent on average $3.46 (SD = 6.4) per day (*p* = 0.02).

### Risky sexual behaviors

As shown in Table 2, the average age at first sexual intercourse for respondents who reported having paid for sex was slightly younger than the age for respondents who reported not having paid for sex (18.8 years ± SD = 1.9 vs. 19.2 years ± SD = 2.4, *p* = 0.08). Respondents who reported having paid for sex had a significantly higher mean number of sexual partners in the past 12 months than those who reported not having paid for sex (4.6±6.8 vs. 1.3±0.9, *p* < 0.001). Moreover, a significantly higher proportion of respondents who reported having paid for sex had an average of more than 4.6 sexual partners in the past 12 months, as compared to respondents who reported not having paid for sex (74.9% vs. 2.8%, *p* < 0.001).

**Table 2:** Sexual behaviors of Cambodian male youth who reported having and having not paid for sex in the last year (*n* = 405)

Sexual behaviors	Having paid for sex (*n* = 334)	Not having paid for sex (*n* = 71)	
	Number (%)	Number (%)	*P*-value^*^
Age at first sex (±SD)	18.8 (±1.9)	19.2 (±2.4)	0.08
Mean sexual partners in the past year (±SD)	4.6 (±6.8)	1.3 (±0.9)	< 0.001
More than 4.6 sexual partners in the past year (average number of sexual partners)			<0.001
Yes	250 (74.9)	2 (2.8)	
No	84 (25.2)	69 (97.2)	
First sexual partner			<0.001
Wife	3 (0.9)	15 (21.1)	
Sweetheart/girlfriend	202 (60.8)	45 (63.4)	
Sex worker in brothel	66 (19.9)	4 (5.6)	
Beer, karaoke, massage or dancing girl	49 (14.8)	4 (5.6)	
Friend	8 (2.4)	2 (2.8)	
Relative	0 (0.0)	1 (1.4)	
Other	4 (1.2)	0 (0.0)	
Ever had a girlfriend			0.69
Yes	306 (91.6)	64 (90.1)	
No	28 (8.4)	7 (9.9)	
Have had a girlfriend or a mistress in the past year			0.89
Yes	232 (75.8)	48 (75.0)	
No	74 (24.2)	16 (25.0)	
Used a condom with most recent girlfriend in the past 3 months (or mistress)			0.29
Always	87 (46.5)	16 (37.2)	
Frequently	5 (2.7)	0 (0.0)	
Sometimes	17 (9.1)	3 (7.0)	
Never	39 (20.9)	9 (20.9)	
Have sweetheart but never had sex with her	39 (20.9)	15 (34.9)	
STI symptoms in the past year			0.56
Yes	20 (6.0)	3 (4.2)	
No	314 (94.0)	68 (95.8)	
Used a condom at last sex with girlfriend			0.58
Yes	128 (67.4)	32 (72.7)	
No	60 (31.6)	11 (25.0)	
Don’t remember	2 (1.1)	1 (2.3)	
Had an HIV test in the last year			
Yes	88 (26.4)	25 (35.2)	0.13
No	246 (73.7)	46 (64.8)	
Paid for sex at age 18 or older			
Yes	469 (95.9)		
No	25 (4.5)		
Sexual partner in the past year:			
Woman working in nightclub/discotheque	83 (24.9)		
Woman working in massage place	42 (12.6)		
Female beer promoter	55 (16.5)		
Female karaoke worker	133 (39.8)		
Woman working in beer garden/restaurant	31 (9.3)		
Female sex worker at brothel/street	149 (44.6)		
Female factory worker	18 (5.4)		
Sweetheart/girl friend	143 (58.6)		
Woman working in nightclub/discotheque	83 (24.9)		
Place you met (see/recruit) the last woman that you paid for sex			
Brothel	67 (20.1)		
Massage parlor	3 (0.9)		
Hotel or guesthouse	202 (60.5)		
Street or park/garden	7 (2.1)		
Karaoke bar	14 (4.2)		
Other	41 (12.3)		
Frequency of condom use in the past 3 months with women that you paid for sex			
Always	188 (88.7)		
Frequently	6 (2.8)		
Sometimes	7 (3.3)		
Never	11 (5.2)		
Number of condom used during the last time that you paid to have sex with a woman			
One	162 (76.4)		
Two or more	50 (23.6)		
You propose using a condom the last time you paid to have sex with a woman			
Yes	161 (75.9)		
No	51 (24.1)		
How much alcohol did you drink the last time you went to a sex-worker?			
A lot (more than 6 small beers or 3 glasses of wine)	105 (31.7)		
Some (3–6 small beers or 2–3 glasses of wine)	54 (16.3)		
A little (1–3 small beers or 1 glass of wine)	31 (9.37)		
No alcohol	141 (42.6)		

*Abbreviations: SD* standard deviation

^*^Chi-square test or Fisher’s exact test was used as appropriate for categorical variables and t-test was used categorical variables

The first sexual partner was most commonly a sweetheart/girlfriend for both respondents who reported having paid for sex (60.8%), and those who reported not having paid for sex (63.4%). The second most frequent first sexual partner among respondents who reported having paid for sex was a sex worker in a brothel (19.9%); conversely, the second most frequent first sexual partner among respondents who reported not having paid for sex was their wife (21.2%). Over one-fifth of respondents who reported not having paid for sex reported their wife as their first sexual partner, whereas only less than 1% of respondents who reported having for sex did the same (21.1% vs. 0.9%, *p* < 0.001).

The majority of both respondents who reported having paid for sex and who reported not having paid for sex had ever had a girlfriend (91.6% and 90.1%, respectively), and reported having a girlfriend or mistress in the past 12 months (75.8% and 75.0%, respectively). Regarding condom use, 46.5% of respondents who reported having paid for sex and 37.2% of respondents who reported not having paid for sex reported always using a condom with their most recent girlfriend in the past 3 months. Moreover, over 65% of both groups (67.4% of respondents who reported having paid for sex and 72.7% of respondents who reported not having paid for sex) reported using a condom the last time they had sex with their current or most recent girlfriend. In the past 12 months, 6.0% of respondents who reported having paid for sex and 4.2% of respondents who reported not having paid for sex reported having at least one STI symptom. About one-third of respondents (26.4% of respondents who reported having paid for sex and 35.2% of respondents who reported not having paid for sex) had been tested for HIV in the past 12 months. No significant difference was found in unadjusted comparisons of risky sexual behaviors among respondents who reported having paid for sex and those who reported not having for sex.

The majority of men you have paid for sex in the last year (95.9%) were 18 years of age or older. The most common types of partners they had sex with in the past 12 months included female karaoke workers (39.8%), female sex worker at a brothel/street (44.6%) and sweetheart/girlfriend (58.6%). The most frequent place where respondents met the last female partner they paid to have sex with was at a hotel or guesthouse (60.5%), followed by a brothel (20.1%). The large majority of respondents (88.7%) reported consistent condom use with women that they paid to have sex with in the past 3 months. While the majority of respondents reported using one condom (76.4%), a large percentage (23.6%) reported using two or more the last time they paid to have sex. However, respondents were not asked if they used the condoms simultaneously or in succession. When asked about the amount of alcohol used at the last time they had paid sex, 42.6% reported that they did not consume alcohol, and 31.7% reported that they drank a lot.

### Factors associated with paying for sex

Table 3 presents factors associated with paying for sex after controlling for other covariates in the multivariate logistic regression model. After adjustment, respondents who reported having paid for sex in the past 12 months remained significantly more likely to be greater than 18 years of age (AOR = 3.6, 95% CI = 1.3–10.6, *p* = 0.02), reside in an urban area (AOR = 2.3, 95% CI = 1.2–4.2, *p* = 0.008), have never been married (AOR = 9.6, 95% CI = 4.3–21.1, *p* < 0.001), spend less than 2.6 USD/day (AOR = 2.2, 95% CI = 1.1–4.4, *p* = 0.02) and have more than 4.6 sexual partners in the past 12 months (AOR = 16.7, 95% CI = 4.7–59.4, *p* < 0.001). Although included in the logistic regression model, occupation, age at first sex, type of first sexual partner, income, educational attainment and HIV testing did not remain significantly associated with paying for sex.

**Table 3:** Factors associated with paying for sex in multiple logistic regression model (*n* = 405)

Variables in the final model	Paying for sex in the past 12 months	
	AOR (95% CI)	*P*-value
Age		
18 years or less	Reference	
Greater than 18 years	3.6 (1.3–10.6)	0.02
Location		
Rural	Reference	
Urban	2.3 (1.2–4.2)	0.008
Marital status		
Married/divorced/separated/living with sexual partner	Reference	
Never married	9.6 (4.3–21.1)	<0.001
Average daily expenditure		
Greater than $2.6 /day	Reference	
Less than $2.6/day	2.2 (1.1–4.4)	0.02
Average number of sexual partners in the past 12 months		
Less than 4.6 partners		
Greater than 4.6 partners	16.7 (4.7–59.4)	<0.001

*Abbreviations*: *AOR* adjusted odds ratio, *CI* confidence interval

Model adjusted for occupation, age at first sex, type of first sexual partner, income, educational attainment and HIV testing

AOR and 95% CI from multivariable logistic regression

## Discussion

This study is one of very few that look at factors associated with paying for sex in Cambodian male youth. The mean age of participants in our study was 21.3 years, much lower than the average age in the two other recent Cambodian studies on the demand side of sex, which were 34.0 and 35.3, respectively [2, 20]. Furthermore, our study specifically sampled most-at-risk young people, therefore 100% of our respondents ranged from 16 to 24 years of age, whereas less than 10% of this age group was included in other reports [2, 20]. The two other studies reported on overlapping regions (major cities in Cambodia) although the NCHADS interviewed 656 men who were exclusively moto-taxi drivers and the Farley interviewed 133 men who had bought sex in the past year.

From the larger sample of 1234, the proportion of respondents that were sexually active was 68.0%, and the proportion that reported paying for sex in the past 12 months was 27.0%. Almost all of those who reported paying for sex (95.9%) were greater than 18 years old. In comparison, of those between 18 and 29 years of age in the Farley study (2012), 44% had paid for sex in the past 12 months [20]. A similar age group (20–24 years old) was also found to be more likely to pay for sex than older age groups (25–35 and >35 years old) in the NCHADS study (2004) [2].

In our study, the reported age of first sex in youth who reported having paid for sex was 18.8 (±1.9), whereas in the other studies it was 20.8 years [20] and 22.1 years [2]. Of all male youth who reported having paid for sex, 34.7% reported their first sex was with a direct female sex worker in a brothel or indirect female sex workers working at a beer garden, karaoke bar or massage parlor. In the other two Cambodian studies, 86.0% [20] and 28.8% [2] reported direct sex workers as their first sexual partner.

Despite the 2008 ban of brothels in Cambodia, 20.1% of male youth who reported having paid for sex in our study reported soliciting sex at brothels; however, this is much less than the 98% of an older population who reported soliciting from brothels in the Farley study (2012) [20]. Of note, 60.5% of male youth in our study also reported soliciting sex at hotels or guesthouses, which have become like brothels where clients commonly meet sex workers.

Despite participating in behaviors that put them at greater risk for contracting HIV, male youth who reported having paid for sex in the past 12 months reported a low HIV testing rates (26.4%). This finding is similar to another study, suggesting that other groups of at-risk young people who participate in high-risk activities may not be accurately perceiving their HIV risk and therefore report low rates of condom use and HIV testing [35]. Youth in general have been shown to have a greater sense of invulnerability than adults, which can affect their perception of their own risk [36]. This risk perception issue may also explain the surprising finding that male youth who were sexually active but had not paid for sex reported higher, although not statistically significant, rates of HIV testing (35.2% vs. 26.4%) [36]. The fact that male youth are participating in risky behaviors such as paying for sex may indicate that their overall risk perception is different than those who are not participating in risky behaviors.

Despite the low HIV testing rates, condom use with sex workers is reportedly high (88.7%), which suggests that, contrary to the previous finding, youth may in fact be perceiving their risk accurately. Condom use with sex workers was encouraged through the national 100% Condom Use Program, which was shown to have increased condom use between sex workers and clients [37]. This program may have introduced response bias in how people answer questions about condom use where they might overstate the regularity with which they use condoms. If youth are indeed using condoms consistently, perhaps that has an effect on their beliefs about the need for an HIV test.

In our study, we found that respondents who resided in an urban location were significantly more likely to report having paid or given gifts to have sex with a woman in the past 12 months. This is supported by other studies from China and Cambodia where the prevalence of selling sex was higher in urban areas than rural areas [38, 39].

We also found that those who reported never having been married were significantly more likely to report having paid or given gifts to have sex with a woman in the past 12 months. In contrast, other studies have suggested that being married increases or has no effect on the likelihood of paying to have sex with a woman. Studies from Western countries show that the majority of men in their studies who had paid for sex were married or had a steady partner [11, 40–42]. A study in Australia showed no significant association between marriage and whether or not they had ever paid for sex [5]. In our study, the number of youth who were married is low, and this may limit our ability to see more detailed explanations for this relationship.

Spending less than $2.6 USD per day (the average daily expense) was significantly associated with having paid or given gifts to have sex with a woman in the past 12 months. In our study, most respondents reported no income, perhaps because many were still in school or dependent on their parents; however, all respondents reported daily expenses. It is not surprising that the relationship between daily expenditures, income and paying for sex is not clearly defined. In our study, paying for sex can be in cash or in kind, which includes small gifts or dinner, for example, which may confound the relationship between income, expenses and paying for sex. We know that paying for sex in Cambodia is not an activity restricted to the middle or high-income segments of society. In fact, men with little income may still pay for sex with cash or in kind [20]. Even men in occupations that do not pay much or young people who do not have jobs are still able to afford to pay for sex [2].

We also found that reporting more than 4.6 sexual partners (the average number of sexual partners) in the past 12 months was significantly associated with paying for sex in the past year. This average was higher than in other studies of men who pay for sex in Cambodia. The Cambodia NCHADS study found that for men who pay for sex, the mean number of partners in the past 12 months was 2.7–3 [2]. In general, having more partners has been associated with paying for sex in studies from Western countries such as the United Kingdom (UK), Sweden and the United States [6, 41, 43, 44]. In the UK, reporting more than five sexual partners over 5 years was strongly associated with paying for sex [6].

### Limitations of the study

There are limitations that are important to consider when interpreting the results of this study. Due to the cross-sectional study design, causal inferences were not possible. In addition, there may be underreporting and over-reporting of the variables that were self-reported. Efforts were made to reduce these biases by collecting responses without links to any identifiable information, and interviews were conducted in confidential locations. Finally, the representativeness of the study sample is limited. The data were collected from only the capital city and seven provinces within Cambodia, which were purposefully selected to include a higher likelihood of having at-risk youth. To address this, probability sampling was used for the sample selection, mapping of hotspots using time-location method was conducted and researchers experienced a very low refusal rate. As a result, we found that the results are representative of at-risk youth within the eight selected city and provinces. Areas of future research may look at men who buy sex from male sex workers, which is a more stigmatized sexual practice and therefore may be associated with greater health risks.

## Conclusions

This study highlights the high proportion of young Cambodian male youth who pay for sex and the potential challenges to addressing this issue in the future. This reflects the fact that, in Cambodia, paying for sex may be considered a cultural norm, a rite of passage and an expression of masculinity or male identity [20, 45]. While the majority of HIV prevention interventions surrounding sex work are aimed at sex workers themselves, targeting the demand side of sex work, particularly the local demand, may be an important next step towards a sustainable prevention [45]. Changing cultural attitudes about manhood and sexuality is one way to address demand, which might be achieved through a combination of public education campaigns, innovative policy changes and enforcement of current laws against buying sex at brothels. Such programs have been successful, for example in Sweden where legal action against sex work clients resulted in a decrease in the number of men who paid for sex [3]. This information can be used to more effectively design and target programs at the highest risk groups. In addition, a harm reduction model may be useful to think about how to address HIV and STI risks for both parties through condom use campaigns and convenient HIV testing sites.

Finally, as in other cultures, Cambodian men express ambivalence about buying sex [20]. Of men who buy sex, the vast majority (89.0%) consider prostitution to be sexual exploitation, and describe guilt, a loss of honor and a decline in Khmer cultural values as a consequence of sex work [20]. This disparity between feelings of guilt or shame and continuing their behaviors represents an opportunity for encouraging behavior change. In our study, we did not ask young men qualitative questions about why they purchase sex or their perceived consequences sex work has on Khmer cultural values. This is an area that future studies on the demand side of sex work should address.

## Abbreviations

AIDS: Acquired immune deficiency syndrome
AOR: Adjusted odds ratio
CDHS: Cambodia Demographic and Health Survey
CI: Confidence interval
FEW: Female entertainment workers
HIV: Human immunodeficiency virus
IOM: International Organization for Migration
MSM: Men who have sex with men
NCHADS: National Center for HIV/AIDS, Dermatology and STD
NECHR: National Ethics Committee for Health Research
OR: Odds ratio
PWID: People who inject drugs
SD: Standard deviation
STD: Sexually transmitted diseases
STIs: Sexually transmitted infections
TG: Transgender
